# (Di­methyl­phosphor­yl)methanaminium nitrate

**DOI:** 10.1107/S1600536813027694

**Published:** 2013-10-16

**Authors:** Claudia M. Bianga, Julia Eggeling, Guido J. Reiss

**Affiliations:** a Institut für Anorganische Chemie und Strukturchemie, Lehrstuhl II: Material- und Strukturforschung, Heinrich-Heine-Universität Düsseldorf, Universitätsstrasse 1, D-40225 Düsseldorf, Germany

## Abstract

In the crystal of the title salt, C_3_H_11_NOP^+^·NO_3_^−^, dicationic inversion dimers are head-to-tail connected by a pair of strong N—H⋯O hydrogen bonds. The resulting graph-set descriptor of this ring system is *R*_2_^2^(10). The nitrate counter-anions connect the dicationic dimers *via* N—H⋯O hydrogen bonds, forming two-dimensional networks in the *bc* plane.

## Related literature

For transition metal complexes of the (dimethylphos­phor­yl)methanamine (*dpma*) ligand, see: Dodoff *et al.* (1990 ▶); Borisov *et al.* (1994 ▶); Trendafilova *et al.* (1997 ▶); Kochel (2009 ▶). For transition metal complexes of the protonated *dpma*H^+^ ligand, see: Reiss (2013*a* ▶,*b* ▶). For simple *dpma*H^+^ salts, see: Reiss & Jörgens (2012 ▶); Lambertz *et al.* (2013 ▶); Buhl *et al.* (2013 ▶); Reiss (2013*c* ▶,*d* ▶). For a definition of the term tecton, see: Brunet *et al.* (1997 ▶). For a definition of the term anti­type, see: Lima-de-Faria *et al.* (1990 ▶). For graph-set theory, see: Etter *et al.*(1990 ▶); Grell *et al.* (2002 ▶). For structures showing an analogous topology, see: Holl & Thewalt (1986 ▶); Reiss (2002 ▶); Reiss & Helmbrecht (2012 ▶).
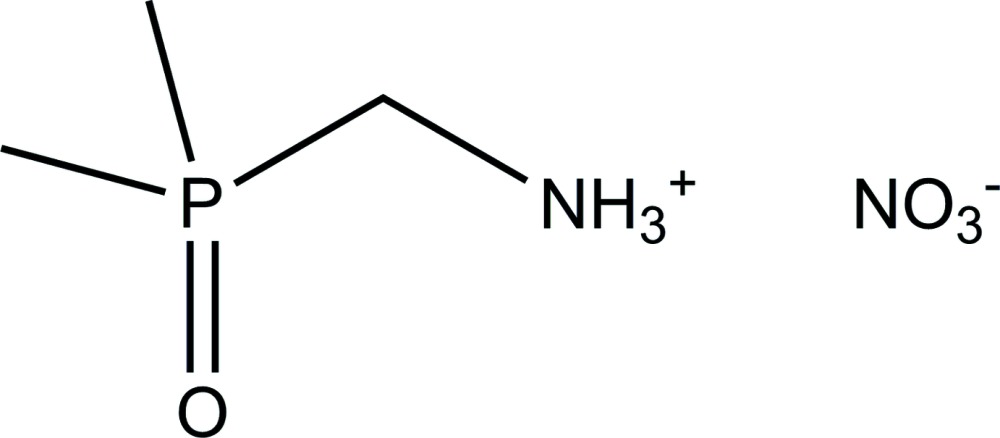


## Experimental

### 

#### Crystal data

C_3_H_11_NOP^+^·NO_3_^−^
*M* *_r_* = 170.11
Monoclinic, 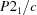

*a* = 8.7718 (3) Å
*b* = 7.9892 (3) Å
*c* = 11.2921 (6) Å
β = 96.581 (4)°
*V* = 786.14 (6) Å^3^
*Z* = 4
Mo *K*α radiation
μ = 0.32 mm^−1^
*T* = 290 K
0.63 × 0.38 × 0.19 mm

#### Data collection

Oxford Diffraction Xcalibur Eos diffractometer
Absorption correction: multi-scan (*CrysAlis PRO*; Oxford Diffraction, 2009 ▶) *T*_min_ = 0.809, *T*_max_ = 1.000
82785 measured reflections
3770 independent reflections
3313 reflections with *I* > 2σ(*I*)
*R*_int_ = 0.032

#### Refinement

*R*[*F* ^2^ > 2σ(*F* ^2^)] = 0.030
*wR*(*F* ^2^) = 0.065
*S* = 1.01
3770 reflections
106 parameters
H atoms treated by a mixture of independent and constrained refinement
Δρ_max_ = 0.40 e Å^−3^
Δρ_min_ = −0.34 e Å^−3^

### 

Data collection: *CrysAlis PRO* (Oxford Diffraction, 2009 ▶); cell refinement: *CrysAlis PRO*; data reduction: *CrysAlis PRO*; program(s) used to solve structure: *SHELXL2013* (Sheldrick, 2008 ▶); program(s) used to refine structure: *SHELXL2013*; molecular graphics: *DIAMOND* (Brandenburg, 2012 ▶); software used to prepare material for publication: *publCIF* (Westrip, 2010 ▶).

## Supplementary Material

Crystal structure: contains datablock(s) I, New_Global_Publ_Block. DOI: 10.1107/S1600536813027694/lh5656sup1.cif

Structure factors: contains datablock(s) I. DOI: 10.1107/S1600536813027694/lh5656Isup2.hkl

Supplementary material file. DOI: 10.1107/S1600536813027694/lh5656Isup3.cml

Additional supplementary materials:  crystallographic information; 3D view; checkCIF report

## Figures and Tables

**Table 1:** Hydrogen-bond geometry (Å, °)

*D*—H⋯*A*	*D*—H	H⋯*A*	*D*⋯*A*	*D*—H⋯*A*
N1—H11⋯O2	0.894 (14)	1.966 (15)	2.8555 (11)	173.1 (12)
N1—H12⋯O3^i^	0.902 (14)	1.979 (14)	2.8784 (12)	174.5 (13)
N1—H13⋯O1^ii^	0.874 (14)	1.888 (14)	2.7493 (10)	168.2 (13)

Symmetry codes: (i) 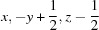; (ii) 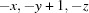.

**Fig. 1.**
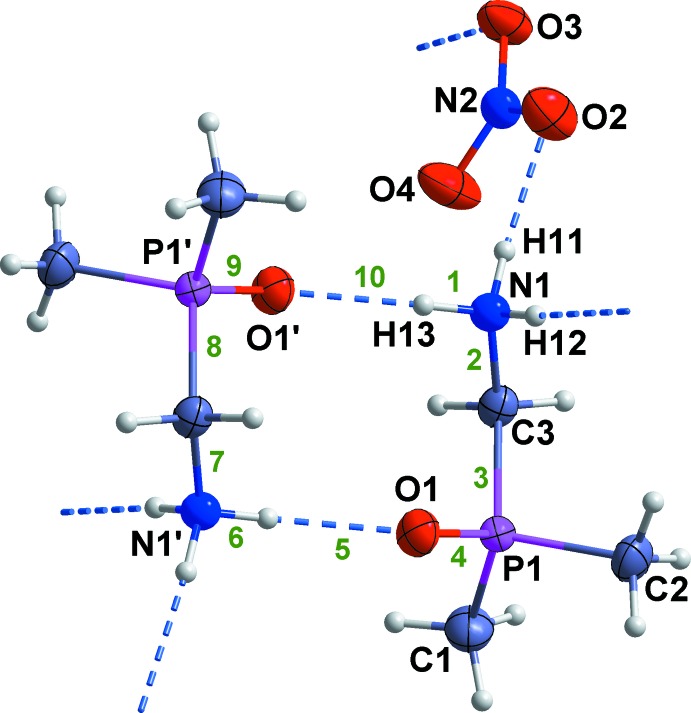
A view of the asymmetric unit of the title structure together with a symmetry related cation and their hydrogen bonds (dashed lines) to adjacent ions. Displacement ellipsoids are drawn at the 45% probability level (' = –x, 1 – y, –z). The green numbers mark the ring size of the first level R22(10) graph-set descriptor for the cyclic dimer consisting of two dpmaH+ cations.)

**Fig. 2.**
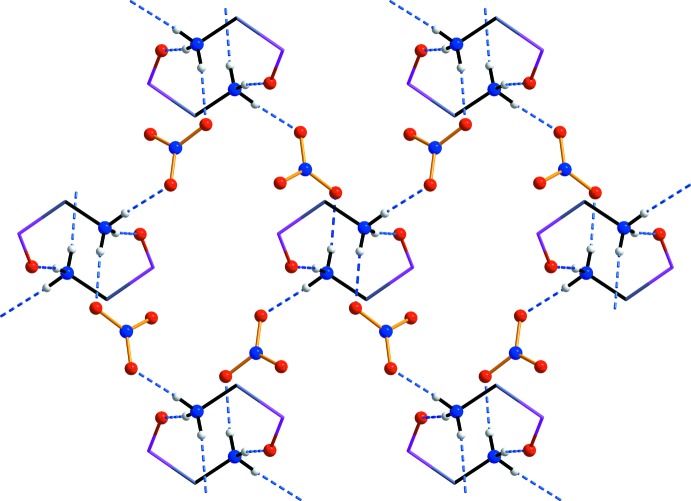
View along [100] on the two-dimensional network of the title structure. Hydrogen bonds are shown as dashed lines.

## supplementary crystallographic information

### 1. Comment

There are several reports which prove the ability of the bidentate *dpma* ligand (*dpma* = (dimethylphosphoryl)methanamine) to coordinate a variety of transition metals (Dodoff *et al.*, 1990; Borisov *et al.*, 1994; Trendafilova *et al.*, 1997; Kochel, 2009). Additionally, two metal complexes containing the *dpma*H^+^ cation have been structurally characterized so far (Reiss, 2013*a*,*b*). For simple *dpma*H^+^ salt structures it has been shown that this tecton (Brunet *et al.*, 1997) can be used to construct hydrogen bonded one-dimensional polymers (Reiss & Jörgens, 2012; Lambertz *et al.*, 2013; Buhl *et al.*, 2013; Reiss, 2013*c*). Only for the double salt (H_3_O)*dpm*aHBr the ions are connected by hydrogen bonds, forming a two-dimensional network (Reiss, 2013*d*)

The title compound *dpma*HNO_3_ crystallizes in the monoclinic space group, *P*2_1_/*c*, with one *dpma*H^+^ cation and one nitrate anion in the asymmetric unit. The angles and bond lengths of both ions are all in the typical ranges. The *dpma*H^+^ cation features the hydrogen bond donor group NH_3_^+^ at the one end and the hydrogen bond accepting group –P=O at the other end. Therefore, this tecton in principle is capable to form connections among other *dpma*H^+^ cations and to counter anions. In the title structure two *dpma*H^+^ cations are connected by two strong, charge supported –NH^+^···O=P– hydrogen bonds (N···O = 2.7493 (10) Å) head to tail forming cyclic dimers (Fig. 1; first level graph-set descriptor: *R*_2_^2^(10); Etter *et al.*,1990; Grell *et al.*, 2002). Moreover, each dimer is associated by charge supported, strong –NH^+^···O≐ N– hydrogen bonds (N···O = 2.8555 (11) Å, 2.8784 (12) Å) to four adjacent nitrate counter anions (Fig. 2). Consequently, each nitrate anion forms two hydrogen bonds to two adjacent dicationic dimers constructing a two-dimensional network in the *bc* plane. The dimers act as a tetradentate hydrogen bond donor. The nitrate anions act as bidentate hydrogen bond acceptors by two of their oxygen atoms.

In three related structures built up by the complex anions [SnCl_6_]^2-^ and [IrCl_6_]^2-^ these anions are tetradentate hydrogen bond acceptors whereas the simple diisopropylammonium (Reiss, 2002; Reiss & Helmbrecht, 2012) and the aminothiodithiazyl ((S_3_N_2_)NH_2_)^+^ (Holl & Thewalt, 1986) counter cations are bidentate hydrogen bond donors. The title structure can be understood as the antitype (Lima-de-Faria *et al.* 1990) of these hexahalogenometallate salts.

### 2. Experimental

The title compound was synthesized by dissolving 1.07 g (10.0 mmol) (dimethylphosphoryl)methanamine in dilute nitric acid (2 ml, 12%) while the mixture was heated. After a few days colourless platelets were obtained by slow evaporation of the solvent at room temperature.

### 3. Refinement

All H-atoms were identified in difference syntheses. Hydrogen atoms at both methyl groups and at the CH_2_ group were idealized and refined using a riding model (AFIX 137 (methyl) and AFIX 23(CH_2_) option of the *SHELXL-2013* program [Sheldrick, 2008; *U*_iso_ = 1.5*U*_eq_(C_methyl_) and 1.2*U*_eq_(C_methylene_)]. The coordinates of the hydrogen atoms at the aminium group were refined freely with individual *U*_iso_ values.

### Crystal data

**Table:**

C_3_H_11_NOP^+^·NO_3_^−^	*F*(000) = 360
*M**_r_* = 170.11	*D*_x_ = 1.437 Mg m^−^^3^
Monoclinic, *P*2_1_/*c*	Mo *K*α radiation, λ = 0.71073 Å
*a* = 8.7718 (3) Å	Cell parameters from 40230 reflections
*b* = 7.9892 (3) Å	θ = 3.8–36.3°
*c* = 11.2921 (6) Å	µ = 0.32 mm^−^^1^
β = 96.581 (4)°	*T* = 290 K
*V* = 786.14 (6) Å^3^	Block, colourless
*Z* = 4	0.63 × 0.38 × 0.19 mm

### Data collection

**Table:**

Oxford Diffraction Xcalibur Eos diffractometer	3770 independent reflections
Radiation source: fine-focus sealed tube	3313 reflections with *I* > 2σ(*I*)
Graphite monochromator	*R*_int_ = 0.032
Detector resolution: 16.2711 pixels mm^-1^	θ_max_ = 36.4°, θ_min_ = 3.8°
ω scans	*h* = −14→14
Absorption correction: multi-scan (*CrysAlis PRO*; Oxford Diffraction, 2009)	*k* = −13→13
*T*_min_ = 0.809, *T*_max_ = 1.000	*l* = −18→18
82785 measured reflections	

### Refinement

**Table:**

Refinement on *F*^2^	Secondary atom site location: difference Fourier map
Least-squares matrix: full	Hydrogen site location: mixed
*R*[*F*^2^ > 2σ(*F*^2^)] = 0.030	H atoms treated by a mixture of independent and constrained refinement
*wR*(*F*^2^) = 0.065	*w* = 1/[σ^2^(*F*_o_^2^) + (0.011*P*)^2^ + 0.3*P*] where *P* = (*F*_o_^2^ + 2*F*_c_^2^)/3
*S* = 1.01	(Δ/σ)_max_ = 0.001
3770 reflections	Δρ_max_ = 0.40 e Å^−^^3^
106 parameters	Δρ_min_ = −0.34 e Å^−^^3^
0 restraints	Extinction correction: *SHELXL2013* (Sheldrick, 2008), Fc^*^=kFc[1+0.001xFc^2^λ^3^/sin(2θ)]^-1/4^
Primary atom site location: structure-invariant direct methods	Extinction coefficient: 0.0296 (13)

### Special details

**Table:**

Experimental. The Raman spectrum was measured using a Bruker MULTIRAM spectrometer (Nd:YAG-Laser at 1064 nm; RT-InGaAs-detector; backscattering geometry); 4000–70 cm^-1^: 3112 (w), 2994 (*s*), 2952 (*m*), 2916 (*s*), 2854 (w), 2834 (w), 2804 (w), 2654 (w), 2625 (w), 2588 (w), 1645 (w), 1615 (w), 1549 (w), 1430 (w), 1407 (w), 1373 (w), 1309 (w), 1155 (*m*), 1126 (w), 1092 (w), 1046 (*s*), 1029 (w), 947 (w), 918 (w), 895 (w), 858 (w), 787 (w), 759 (w), 726 (*m*), 662 (*s*), 456 (w), 369 (w), 314 (*m*), 295 (w), 268 (w), 238 (w), 137 (w), 121 (*m*), 95 (*s*), 73 (*s*). – IR spectroscopic data were recorded on a Digilab FT3400 spectrometer using a MIRacle ATR unit (Pike Technologies); 4000–560 cm^-1^: 3439 (w), 2993 (*s*), 2946 (*s*), 2915 (*s*), 2885 (*s*), 2830 (*s*), 2749 (*m*), 2722 (*m*), 2651 (*m*), 2625 (*m*), 2403 (w), 2066 (w), 1757 (w), 1638 (*m*), 1551 (*m*), 1432 (*m*), 1420 (*m*), 1405 (*m*), 1346 (*s*), 1330 (*s*), 1306 (*s*), 1297 (*s*), 1157 (*s*), 1119 (*m*), 1088 (*s*), 1045 (w), 1029 (w), 944 (*s*), 914 (*m*), 889 (*s*), 855 (*m*), 826 (*m*), 784 (*m*), 756 (*m*), 723 (w), 714 (w), 657 (w).
Geometry. All e.s.d.'s (except the e.s.d. in the dihedral angle between two l.s. planes) are estimated using the full covariance matrix. The cell e.s.d.'s are taken into account individually in the estimation of e.s.d.'s in distances, angles and torsion angles; correlations between e.s.d.'s in cell parameters are only used when they are defined by crystal symmetry. An approximate (isotropic) treatment of cell e.s.d.'s is used for estimating e.s.d.'s involving l.s. planes.

### Fractional atomic coordinates and isotropic or equivalent isotropic displacement parameters (Å^2^)

**Table:**

	*x*	*y*	*z*	*U*_iso_*/*U*_eq_	
P1	0.16112 (2)	0.75856 (3)	0.06006 (2)	0.02633 (6)	
O1	0.04435 (8)	0.70859 (9)	−0.04036 (6)	0.03792 (15)	
N1	0.24474 (9)	0.42711 (10)	0.08323 (7)	0.03066 (14)	
H11	0.2939 (15)	0.3482 (18)	0.1290 (12)	0.050 (4)*	
H12	0.2989 (15)	0.4449 (17)	0.0211 (13)	0.051 (4)*	
H13	0.1553 (16)	0.3832 (18)	0.0591 (12)	0.052 (4)*	
C1	0.09272 (13)	0.90021 (14)	0.16403 (10)	0.0431 (2)	
H1A	0.0774	1.0085	0.1278	0.065*	
H1B	0.1667	0.9085	0.2333	0.065*	
H1C	−0.0027	0.8598	0.1869	0.065*	
C2	0.32841 (11)	0.84891 (13)	0.00985 (10)	0.0400 (2)	
H2A	0.3758	0.7689	−0.0377	0.060*	
H2B	0.3994	0.8799	0.0774	0.060*	
H2C	0.3000	0.9465	−0.0371	0.060*	
C3	0.22309 (10)	0.57989 (11)	0.15315 (7)	0.02919 (15)	
H3A	0.3190	0.6073	0.2007	0.035*	
H3B	0.1473	0.5578	0.2073	0.035*	
O2	0.41973 (10)	0.17393 (12)	0.21553 (7)	0.0511 (2)	
O3	0.41189 (9)	0.03797 (10)	0.37965 (7)	0.04730 (19)	
O4	0.26575 (11)	0.25210 (12)	0.33988 (9)	0.0596 (2)	
N2	0.36518 (9)	0.15533 (10)	0.31187 (7)	0.03293 (15)	

### Atomic displacement parameters (Å^2^)

**Table:**

	*U*^11^	*U*^22^	*U*^33^	*U*^12^	*U*^13^	*U*^23^
P1	0.02420 (9)	0.02590 (9)	0.02829 (10)	−0.00258 (7)	0.00046 (7)	0.00021 (7)
O1	0.0351 (3)	0.0393 (3)	0.0364 (3)	−0.0046 (3)	−0.0083 (3)	−0.0003 (3)
N1	0.0296 (3)	0.0285 (3)	0.0324 (3)	0.0006 (3)	−0.0028 (3)	0.0034 (3)
C1	0.0432 (5)	0.0381 (5)	0.0488 (5)	0.0000 (4)	0.0091 (4)	−0.0112 (4)
C2	0.0363 (4)	0.0384 (5)	0.0463 (5)	−0.0080 (4)	0.0093 (4)	0.0071 (4)
C3	0.0303 (4)	0.0314 (4)	0.0255 (3)	−0.0033 (3)	0.0015 (3)	0.0026 (3)
O2	0.0497 (4)	0.0641 (5)	0.0410 (4)	0.0132 (4)	0.0115 (3)	0.0187 (4)
O3	0.0484 (4)	0.0474 (4)	0.0467 (4)	0.0145 (3)	0.0078 (3)	0.0202 (3)
O4	0.0608 (5)	0.0584 (5)	0.0621 (5)	0.0296 (4)	0.0177 (4)	0.0120 (4)
N2	0.0290 (3)	0.0338 (4)	0.0346 (3)	0.0006 (3)	−0.0021 (3)	0.0046 (3)

### Geometric parameters (Å, º)

**Table:**

P1—O1	1.4927 (7)	C1—H1C	0.9600
P1—C1	1.7834 (10)	C2—H2A	0.9600
P1—C2	1.7851 (9)	C2—H2B	0.9600
P1—C3	1.8186 (9)	C2—H2C	0.9600
N1—C3	1.4777 (12)	C3—H3A	0.9700
N1—H11	0.894 (14)	C3—H3B	0.9700
N1—H12	0.902 (14)	O2—N2	1.2465 (11)
N1—H13	0.874 (14)	O3—N2	1.2494 (10)
C1—H1A	0.9600	O4—N2	1.2336 (11)
C1—H1B	0.9600		
			
O1—P1—C1	114.62 (5)	H1B—C1—H1C	109.5
O1—P1—C2	112.60 (5)	P1—C2—H2A	109.5
C1—P1—C2	107.66 (5)	P1—C2—H2B	109.5
O1—P1—C3	111.27 (4)	H2A—C2—H2B	109.5
C1—P1—C3	102.61 (5)	P1—C2—H2C	109.5
C2—P1—C3	107.38 (5)	H2A—C2—H2C	109.5
C3—N1—H11	110.9 (9)	H2B—C2—H2C	109.5
C3—N1—H12	113.4 (9)	N1—C3—P1	112.81 (6)
H11—N1—H12	107.4 (12)	N1—C3—H3A	109.0
C3—N1—H13	109.4 (9)	P1—C3—H3A	109.0
H11—N1—H13	104.7 (12)	N1—C3—H3B	109.0
H12—N1—H13	110.7 (12)	P1—C3—H3B	109.0
P1—C1—H1A	109.5	H3A—C3—H3B	107.8
P1—C1—H1B	109.5	O4—N2—O2	120.16 (8)
H1A—C1—H1B	109.5	O4—N2—O3	120.34 (9)
P1—C1—H1C	109.5	O2—N2—O3	119.50 (8)
H1A—C1—H1C	109.5		
			
O1—P1—C3—N1	40.98 (7)	C2—P1—C3—N1	−82.67 (7)
C1—P1—C3—N1	164.01 (6)		

### Hydrogen-bond geometry (Å, º)

**Table:**

*D*—H···*A*	*D*—H	H···*A*	*D*···*A*	*D*—H···*A*
N1—H11···O2	0.894 (14)	1.966 (15)	2.8555 (11)	173.1 (12)
N1—H12···O3^i^	0.902 (14)	1.979 (14)	2.8784 (12)	174.5 (13)
N1—H13···O1^ii^	0.874 (14)	1.888 (14)	2.7493 (10)	168.2 (13)

Symmetry codes: (i) *x*, −*y*+1/2, *z*−1/2; (ii) −*x*, −*y*+1, −*z*.
